# Ergothioneine Improves the Quality of Boar Sperm During *In Vitro* Liquid Preservation by Regulating Mitochondrial Respiratory Chain

**DOI:** 10.3390/ani15101450

**Published:** 2025-05-17

**Authors:** Qing Guo, Xue Liu, Yang Li, Ye Cheng, Jingchun Li

**Affiliations:** College of Animal Science and Veterinary Medicine, Heilongjiang Bayi Agricultural University, Daqing 163319, China; lx729c@126.com (X.L.); li03122025@126.com (Y.L.); 15945036360@163.com (Y.C.); lljchun112@163.com (J.L.)

**Keywords:** boar semen liquid preservation, sperm quality, ergothioneine, mitochondrial electron respiratory chain, rotenone

## Abstract

Porcine artificial insemination primarily utilizes liquid-preserved (17 °C) semen; however, the quality of sperm diminishes progressively with extended preservation time. Ergothioneine (EGT) is a mitochondria-targeting antioxidant. Therefore, this study aimed to analyze the effect of various concentrations of EGT (0, 0.15, 0.3, and 0.6 mM) on the quality of boar sperm during *in vitro* liquid preservation and elucidate the underlying mechanisms of the mitochondrial electron respiratory chain inhibitor ROT. The results demonstrated that the addition of 0.3 mM EGT to the modified Modena extender significantly improved sperm motility and kinetic parameters, as well as mitochondrial membrane potential (MMP), adenosine triphosphate (ATP), antioxidant capacity, and the integrity of both the sperm plasma membrane and acrosome. Additionally, ROT significantly inhibited sperm motility, kinetic parameters, MMP, ATP levels, antioxidant capacity, and sperm integrity of the plasma membrane and acrosome. However, these adverse effects could be partially mitigated by the addition of 0.3 mM EGT. In conclusion, the novel findings of this study indicated that EGT plays a crucial role in protecting sperm from oxidative damage by regulating the mitochondrial electron respiratory chain, suggesting that the use of EGT is a promising approach for enhancing the *in vitro* liquid preservation efficiency of boar semen at 17 °C.

## Simple Summary

During liquid preservation of boar semen at 17 °C, the quality of spermatozoa declines primarily due to the excessive production of reactive oxygen species (ROS), which results from damage to the mitochondrial respiratory chain as the preservation time extends. Research has shown that ergothioneine (EGT) possesses significant antioxidant properties, primarily targeting mitochondria. This study demonstrates that EGT significantly enhances the efficiency of boar semen preservation at room temperature, with an optimal concentration of 0.3 mM. Further investigation using the mitochondrial respiratory chain inhibitor rotenone (ROT) revealed that EGT can significantly ameliorate ROT-induced damage to boar spermatozoa. This finding suggests that EGT may enhance the efficiency of boar semen preservation at room temperature by modulating the mitochondrial respiratory chain.

## 1. Introduction

Currently, the modern porcine farming industry primarily relies on artificial insemination (AI), with over 99% of semen preserved at 17 °C [1]. Approximately 85% of boar semen can be preserved at room temperature; however, pig farms generally utilize semen that has been stored at this temperature for no longer than three days [2]. A significant factor contributing to this is the propensity for reactive oxygen species (ROS) to be generated during the preservation process, which can damage sperm and reduce preservation efficiency. Therefore, adding exogenous antioxidants is an effective method to enhance the *in vitro* liquid preservation efficiency of pig semen and other species [3].

In mammalian sperm, the mitochondrial electron transport chain serves as the principal source of ROS. Under normal physiological conditions, around 0.1% to 0.2% of the electrons in the respiratory chain leak and react with molecular oxygen, resulting in the formation of a superoxide anion (O^2−^) [4]. This electronic leakage occurs at various sites within the respiratory chain, including the binding sites of respiratory chain complexes I and III. In states of oxidative stress, these regions of the sperm mitochondria can lead to significant electron leakage, producing elevated levels of ROS. Moderate concentrations of ROS in sperm promote capacitation [5], hyperactivation [6], and acrosomal reaction [7], while excessive ROS will result in lipid peroxidation [8], DNA damage [9], and apoptosis [10]. Generally, different ingredients such as antibiotics [11] and antioxidants [12] were added to the extender to suppress sperm metabolism, slow down sperm movement, reduce energy consumption, and achieve long-term preservation. In sperm, specific inhibitors targeting complex I and complex III of the electron transport chain will lead to an increase in the level of ROS at these sites. Consequently, compounds such as rotenone (ROT) are frequently utilized to modulate the mitochondrial electron transport chain [13].

Ergothioneine (EGT) is a naturally occurring antioxidant that is present in various tissues and organs, including the kidneys, red blood cells, and liver. It plays a significant role in anti-oxidation, anti-apoptosis, and the regulation of inflammation within the body [14]. Additionally, EGT is also found in mammalian seminal plasma, with a concentration of approximately 582 µM/mL in porcine seminal plasma [15]. Numerous studies have demonstrated that the protective effects of EGT on cells are primarily through the regulation of mitochondrial function. EGT regulates several signaling pathways and genes associated with mitochondrial metabolism, including protein kinase B (AKT) [16], mammalian target of rapamycin (mTORC1) [17], ribosomal protein S6 kinase, polypeptide1 (S6K1) [17], and mitogen-activated protein kinase/extracellular signal-regulated kinase (MAPK/ERK) [16].

We hypothesize that the addition of EGT can improve the liquid preservation efficiency in boar; therefore, the aim of this study was to investigate whether EGT enhances the liquid preservation efficiency of boar semen at 17 °C *in vitro* by modulating the mitochondrial respiratory chain. This research provided a valuable reference for the applications of EGT in boar sperm *in vitro* liquid preservation and a theoretical basis for EGT in treating mitochondrial damage-related diseases.

## 2. Materials and Methods

### 2.1. Chemicals

EGT (≥98% HPLC) was bought from Chengdu Preferred Biotechnology Co., Ltd. (Chengdu, China). Superoxide dismutase (SOD, A001-3-2), glutathione (GSH, A005-1-2), malondialdehyde (MDA, A003-1-2), and adenosine triphosphate (ATP, A095-1-1) kits were obtained from Nanjing Jiancheng Bioengineering Institute (Nanjing, China). All other chemicals used in this study were of analytical reagent grade. Unless otherwise specified, all chemicals were purchased from Sigma Chemical (St. Louis, MO, USA).

### 2.2. Semen Collection and Treatment

Six large, white, breeding boars aged 2–3 years were utilized for semen collection and housed in a clean environment maintained at 24 ± 1 °C, with feed and water provided ad libitum. All experiments were conducted in accordance with the guidelines set by the Management Committee of the Experimental Animal Center (MCEAC) at Heilongjiang Bayi Agricultural University. Semen was collected using the gloved-hand technique and transported to the laboratory within 1 h at 37 °C. The samples were then equilibrated at room temperature on a sterile operating table for 30 min. A 10 μL aliquot of fresh semen was placed on a pre-warmed slide and then on a heated plate at 37 °C to assess sperm motility using the computer-assisted sperm analysis (CASA) system (Songjingtianlun Biotechnology, Nanning, China) [18]. Only samples exhibiting sperm with progressive motility above 75% were used for subsequent experiments.

### 2.3. Experimental Design

Fresh semen was collected in a centrifuge tube and centrifuged at 750× *g* for 3 min at 17 °C. The supernatant was removed, and the sperm pellet was resuspended at a concentration of 1 × 10^8^ cells/mL in a modified Modena extender [19] containing various concentrations of EGT (0, 0.15, 0.3, and 0.6 mM). The modified Modena extender included 46.64 mmol/L Tris, 152.64 mmol/L glucose, 15.09 mmol/L citric acid, 11.90 mmol/L sodium bicarbonate, 26.74 mmol/L sodium citrate, 1 million U/L streptomycin, 6.98 mmol/mL EDTA-2Na·H_2_O, 1 million U/L penicillin, and 4.00 g/L BSA [19]. Subsequently, all extended sperm samples were preserved in an incubator at 17 °C. Sperm motility parameters and kinetic parameters were evaluated at days 1, 3, 5, and 7, aiming to identify the optimal concentration of EGT treatment. Following this, the sperm plasma membrane integrity, acrosome integrity, antioxidant capacity, mitochondrial membrane potential (MMP), and ATP level were assessed at days 1, 3, 5, and 7 in both the control group and the optimal concentration EGT group. Subsequently, after semen preservation for 5 days, ROS levels in boar sperm were measured to determine the effects of EGT on the mitochondrial respiratory chain. Additionally, boar semen was treated with varying concentrations of ROT (0, 0.1, 1, and 10 μM) for a duration of 3 h to identify the concentration of ROT that adversely affects sperm motility and kinetic parameters [20]. Concurrently, the optimal concentrations of EGT and the ROT that negatively impact sperm motility and kinetic parameters were jointly applied to evaluate whether EGT enhances the efficacy of porcine semen preservation at 17 °C by modulating the mitochondrial respiratory chain.

### 2.4. Measurement of Sperm Motility and Kinetic Parameters

The CASA system parameters were set to 30 frames/s to measure sperm motility and kinetic parameters [21]. A 10 μL sperm sample was placed onto a chamber slide with a coverslip and preheated for 15 min at 37 °C. Sperm kinetic parameters, including total motility, progressive motility, average straight-line velocity (VSL), average curvilinear velocity (VCL), beat-cross frequency (BCF), and average path velocity (VAP), were measured on days 1, 3, 5, and 7. Motility was primarily indicated by the percentage of sperm with a straightness of path (STR) greater than 75% and VSL exceeding 25 μm/s. The sperm samples were analyzed using the accompanying software. Each sample was randomly selected across 5 fields, with at least 200 sperm recorded in each field, and the process was repeated three times.

### 2.5. Measurement of Sperm Mitochondrial Membrane Potential

The MMP of sperm was assessed using the JC-1 fluorescent probe and propidium iodide (PI), following a previously established protocol with appropriate modifications [22]. In summary, 100 μL of the sperm sample was mixed with 400 μL of an isotonic buffer diluent containing 1 mM JC-1 and 5 mM PI, and incubated for 30 min at 37 °C. Subsequently, 10 μL of the sperm sample was placed on a slide with a coverslip, photographed using an inverted fluorescence microscope (Mshot Photoelectric Technology, Guangzhou, China), and analyzed with the accompanying software (Olympus, Tokyo, Japan). For each sample, the sperm count exceeded 200. Red fluorescence observed in sperm indicates a high MMP (hMMP), while green fluorescence shows medium to low MMP.

### 2.6. Measurement of Sperm Plasma Membrane Integrity

The method for staining the sperm plasma membrane was adapted from previous studies with appropriate modifications [23]. A 250 μL semen sample was centrifuged at 1200 RPM for 2 min, after which the supernatant was discarded. Concurrently, the HEPES buffer containing 10% BSA and 5 μL of SYBR-14 dye working solution (0.1 mmol/mL) was added, followed by incubation at 37 °C for 5 min. Subsequently, 5 μL of the PI working solution (0.1 mg/mL) was introduced, and the sample was incubated further at 37 °C for 10 min. The samples were examined under a fluorescence microscope in the dark, with 5 areas per group analyzed, ensuring that no fewer than 200 sperm were assessed.

### 2.7. Measurement of Sperm Acrosome Integrity

Sperm acrosome integrity was assessed by fluorescein peanut agglutinin isothiocyanate (FITC-PNA) as reported by Aboagla et al. [24] with appropriate modifications. First, 30 μL of the sperm sample was mounted on a clean slide and allowed to air dry naturally. The sample was then fixed with anhydrous methanol for 10 min and air dried again. Following this, 10 μL of the FITC-PNA (100 μg/mL) working solution was applied to the sample, incubated at 37 °C for 10 min, and subsequently washed three times with phosphate-buffered saline (PBS). Following this, fluorescence microscopy was utilized to capture images, allowing for the calculation of sperm plasma acrosome integrity.

### 2.8. Measurement of Sperm Total Antioxidant Capacity Activity, H_2_O_2_, and MDA Levels

The detection of sperm total antioxidant capacity (T-AOC) activity, as well as the levels of hydrogen peroxide (H_2_O_2_) and ATP levels, was conducted according to a previous study with appropriate modification [19]. A spectrophotometer (PERSEE, Beijing, China) was utilized for detection, with the wavelengths set at 593 nM, 415 nM, and 450 nM, respectively.

### 2.9. Measurement of Sperm ROS Levels

ROS were analyzed using the MitoSOX™ Red Assay Kit as reported by Zhu et al. [25]. Semen samples from each treatment group were centrifuged and resuspended in 500 μL of the working solution, followed by incubation at 37 °C in the dark for 10 min. The sperm samples were then centrifuged and washed three times with PBS. The stained sperm samples were resuspended in PBS to achieve a density of 10^6^ sperm/mL and analyzed using a flow cytometer (Beckman Coulter, Brea, CA, USA). The fluorescence signal intensity of the sperm was assessed in the red fluorescence channel at 488/510 nm, with 10,000 sperm detected for each sample.

### 2.10. Statistical Analysis

All data from each experiment were used in the one-sample Kolmogorov–Smirnov’s test to assess normality. In instances where the data did not conform to a normal distribution, an arcsine-transformation was applied to the variables prior to analysis, and normality was re-evaluated using the one-sample Kolmogorov–Smirnov test for that specific parameter. Furthermore, the data from each experiment were assessed for homogeneity of variance utilizing Levene’s test. The results of the *T*-test indicated that the replicated data from each experiment were homogeneous. Subsequently, all data were analyzed using one-way ANOVA (SPSS 17.0, IBM, Armonk, NY, USA) with STATVIEW 5.0 (Abacus Concepts, Berkeley, CA, USA). *p* < 0.05 was considered a significant difference. All histograms were obtained using Prism 6.0 (GraphPad, San Diego, CA, USA). All data were expressed as the mean ± SEM.

## 3. Results

### 3.1. Effects of Different Concentrations of EGT on Boar Sperm Motility and Kinetic Parameters

The effects of different concentrations of EGT on the sperm motility and kinetic parameters of boar sperm during liquid preservation at 17 °C are presented in Table 1.

On day 1, treatments with 0.15 mM and 0.6 mM EGT did not show a significant difference in the total motility of sperm compared to the control group (*p* > 0.05). In contrast, treatment with 0.3 mM EGT significantly improved the total motility of sperm compared to the control group (*p* < 0.05). Furthermore, on days 3, 5, and 7, the 0.6 mM EGT treatment did not significantly affect sperm total motility relative to the control group (*p* > 0.05), whereas both the 0.15 and 0.3 mM EGT treatments significantly enhanced the total motility of sperm (*p* < 0.05).

Moreover, compared to the control group, 0.3 mM EGT significantly enhanced the progressive motility of sperm on days 1 and 7, while on days 3 and 5, 0.15 mM, 0.3 mM, and 0.6 mM EGT all significantly improved the progressive motility of sperm (*p* < 0.05).

Additionally, on days 1 and 3, the 0.15 and 0.6 mM EGT groups did not significantly affect sperm VAP, VCL, and VSL compared to the control group (*p* > 0.05); however, the 0.3 mM EGT group significantly improved sperm VAP, VCL, and VSL (*p* < 0.05). Furthermore, on days 5 and 7, 0.15 mM EGT treatment had no significant effects on sperm VAP and VCL compared to the control group (*p* > 0.05), while both 0.3 and 0.6 mM EGT treatments significantly enhanced sperm VAP and VCL (*p* < 0.05). Moreover, on days 5 and 7, 0.15, 0.3, and 0.6 mM EGT significantly improved the VSL of boar sperm compared to the control group (*p* < 0.05). Consequently, we selected 0.3 mM EGT for further investigation.

### 3.2. Effects of EGT on Sperm Acrosome and Plasma Membrane Integrity, MMP, and ATP Level

**Figure 1:**
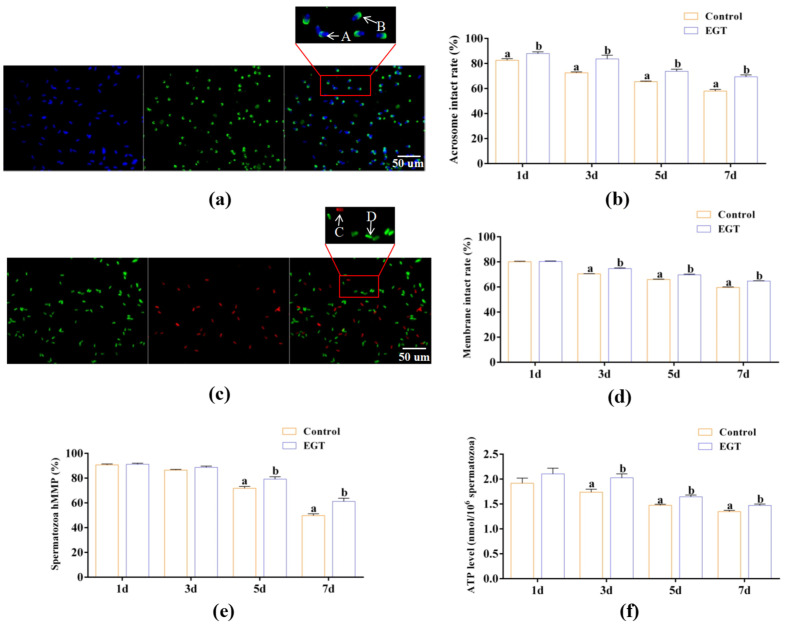
Effects of EGT on sperm acrosome and plasma membrane integrity, MMP, and ATP level. (**a**) FITC-PNA staining for sperm acrosome integrity. Among them, A represents sperm acrosome damage, while B indicates intact sperm acrosome. Scale bars: 50 μm. (**b**) Sperm acrosome intact rate (%). (**c**) SYBR-14/PI staining for sperm plasma membrane integrity. Among them, C denotes sperm plasma membrane damage, whereas D indicates intact sperm plasma membrane. Scale bars: 50 μm. (**d**) Sperm plasma membrane intact rate (%). (**e**) Sperm hMMP rate (%). (**f**) Sperm ATP level. The concentration utilized for EGT is 0.3 mM. *n* = 3, Different letters indicate significant difference (*p* < 0.05) between the control group and the EGT group on days 1, 3, 5, and 7.

The effects of EGT on sperm acrosome and plasma membrane integrity, MMP, and ATP level are shown in Figure 1.

In terms of sperm acrosome and plasma membrane integrity, on day 1, 0.3 mM EGT significantly enhanced sperm acrosome integrity compared to the control group (Figure 1a,b, *p* < 0.05); however, it had no effect on sperm plasma membrane integrity (Figure 1c,d, *p* > 0.05). Moreover, on days 3, 5, and 7, 0.3 mM EGT significantly improved both acrosome integrity (Figure 1a,b, *p* < 0.05) and plasma membrane integrity (Figure 1c,d, *p* < 0.05).

In terms of sperm MMP and ATP levels, on days 1 and 3, EGT did not significantly affect the MMP in sperm compared to the control group (Figure 1e, *p* > 0.05); however, on days 5 and 7, it significantly enhanced the MMP of sperm (Figure 1e, *p* < 0.05). Additionally, on day 1, 0.3 mM EGT did not demonstrate a significant effect on ATP levels of sperm in comparison to the control group (Figure 1f, *p* > 0.05). In contrast, on days 3, 5, and 7, EGT significantly improved ATP levels in sperm (Figure 1f, *p* < 0.05).

### 3.3. Effects of EGT on Boar Sperm T-AOC Activity and H_2_O_2_ Levels

**Figure 2:**
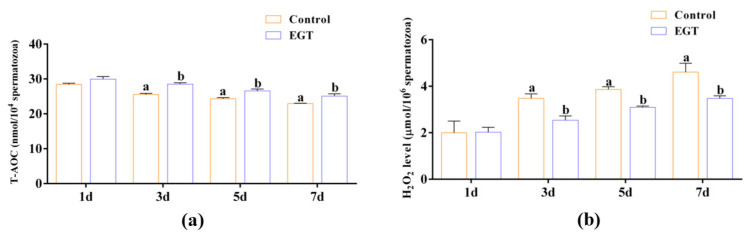
Effects of EGT on boar sperm T-AOC activity (**a**) and H_2_O_2_ level (**b**). The concentration utilized for EGT is 0.3 mM. *n* = 3. Different letters indicate significant difference (*p* < 0.05) between the control group and the EGT group on days 1, 3, 5, and 7.

To investigate the effect of EGT on the antioxidant capacity of boar sperm, we measured the T-AOC and H_2_O_2_ levels in sperm. On day 1, the results indicated that, compared to the control group, EGT did not significantly affect the T-AOC activity (Figure 2a, *p* > 0.05) and H_2_O_2_ (Figure 2b, *p* > 0.05) levels. However, on days 3, 5, and 7, EGT significantly enhanced the T-AOC activity (Figure 2a, *p* < 0.05) and reduced H_2_O_2_ (Figure 2a, *p* < 0.05) levels in the sperm (Figure 2b, *p* < 0.05).

**Figure 3:**
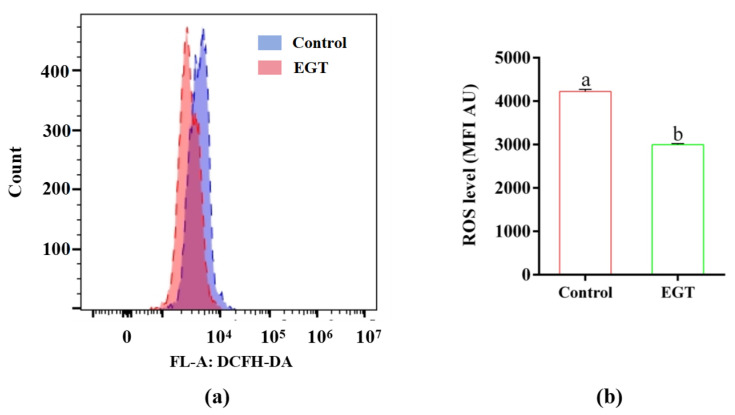
Effects of EGT on boar sperm ROS levels. (**a**) Sperm flow cytometry detected ROS. (**b**) Sperm ROS levels. The concentration utilized for EGT is 0.3 mM. *n* = 3. Different letters indicate significant difference (*p* < 0.05) between the control group and the EGT group.

Additionally, damage to the mitochondrial respiratory chain leads to increased levels of ROS during boar semen liquid preservation. Our results indicated that, in comparison to the control group, 0.3 mM EGT significantly reduced sperm ROS levels (Figure 3a,b, *p* < 0.05).

### 3.4. Effects of ROT on Boar Sperm Motility and Kinetic Parameters

To further investigate the potential mechanism by which EGT enhances the liquid preservation efficiency of boar semen, we treated the semen with various concentrations of the mitochondrial respiratory chain inhibitor ROT (0, 0.1 µM, 1 µM, and 10 µM) for a duration of 3 h. The results are shown in Table 2.

Compared to the control group, treatment with 0.1 µM ROT did not significantly affect the total motility, progressive motility, VAP, VCL, and BCF of sperm (Table 2, *p* > 0.05). Meanwhile, 1 µM ROT treatment significantly decreased the progressive motility, VSL, and BCF of sperm (Table 2, *p* < 0.05), while having no significant impact on sperm total motility (Table 2, *p* > 0.05). In contrast, 10 µM ROT significantly decreased the total motility, progressive motility, VAP, VSL, VCL, and BCF of sperm during the *in vitro* liquid preservation of boar semen at 17 °C (Table 2, *p* < 0.05). Consequently, we selected 10 µM ROT for further investigation.

### 3.5. EGT Improved the Motility and Kinetic Parameters in ROT-Treated Sperm

EGT improved the sperm motility and kinetic parameters in ROT-treated sperm, as shown in Table 3.

Compared to the control group, treatment with 10 µM ROT significantly decreased the total motility, progressive motility, VAP, VSL, VCL, and BCF of sperm (Table 3, *p* < 0.05). In contrast, 0.3 mM EGT significantly restored these changes compared to the ROT treatment group (Table 3, *p* < 0.05), although total motility remained lower than that of the control group.

### 3.6. EGT Improved the Acrosome and Plasma Membrane Integrity, MMP, and ATP Levels in ROT-Treated Sperm

**Figure 4:**
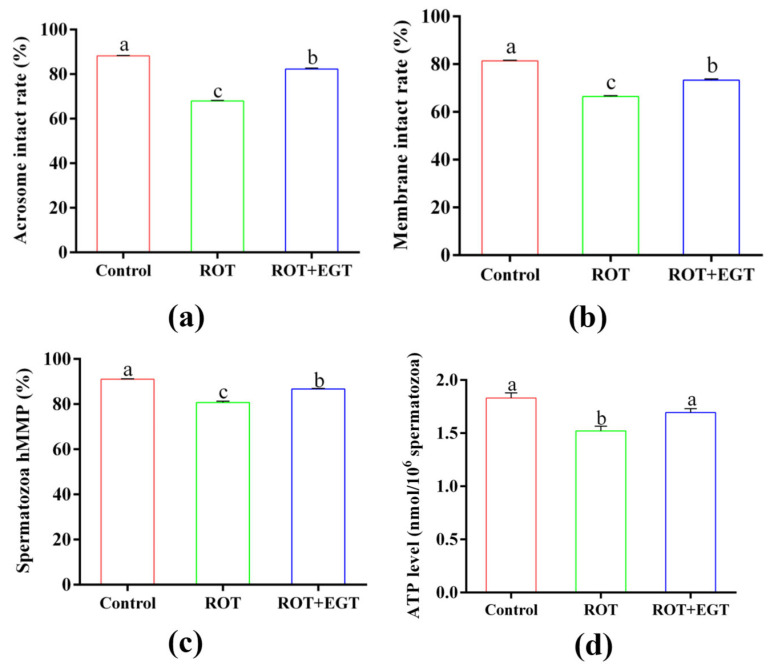
EGT improved the acrosome and plasma membrane integrity, MMP levels, and ATP levels in ROT-treated sperm. (**a**) Sperm acrosome intact rate (%). (**b**) Sperm plasma membrane intact rate (%). (**c**) Sperm hMMP rate (%). (**d**) Sperm ATP level. The concentration utilized for EGT is 0.3 mM and ROT is 10 μM. *n* = 3. Different letters indicate significant difference (*p* < 0.05) between the control group and the EGT group at 3h.

EGT improved the sperm acrosome and plasma membrane integrity, MMP levels, and ATP levels in ROT-treated sperm, as shown in Figure 4.

Compared to the control group, 10 µM ROT significantly decreased the integrity of acrosome (Figure 4a, *p* < 0.05) and the plasma membrane (Figure 4b, *p* < 0.05). However, treatment with 0.3 mM EGT partially reversed these effects (Figure 4ab, *p* < 0.05). Additionally, compared to the control group, treatment with 10 µM ROT significantly decreased the hMMP in sperm, while treatment with 0.3 mM EGT partially restored this alteration (Figure 4c, *p* < 0.05). Furthermore, 10 µM ROT significantly reduced ATP levels in sperm relative to the control group, whereas 0.3 mM EGT significantly reversed this change (Figure 4d, *p* < 0.05), resulting in no significant difference between the control and EGT-treated groups (Figure 4d, *p* > 0.05).

### 3.7. EGT Improved the Antioxidant Capability in ROT-Treated Sperm

**Figure 5:**
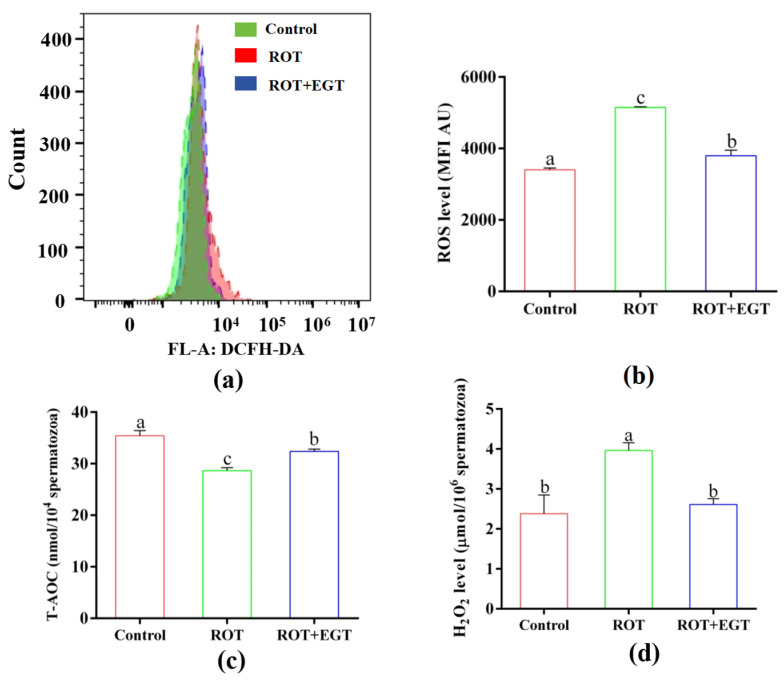
EGT improved the antioxidant capability in ROT-treated sperm. (**a**) Sperm flow cytometry detected ROS. (**b**) ROS levels. (**c**) T-AOC activity. (**d**) H_2_O_2_ level. The concentration utilized for EGT is 0.3 mM and ROT is 10 μM. *n* = 3. Different letters indicate significant difference (*p* < 0.05) between the control group and the EGT group at 3h.

EGT improved the antioxidant capability in ROT-treated sperm, as shown in Figure 5.

Damage to the mitochondrial respiratory chain leads to electron leakage, resulting in increased levels of ROS and oxidative stress. The results demonstrated that treatment with 10 µM ROT significantly increased ROS levels in sperm compared to the control group, while 0.3 mM EGT significantly restored these levels (Figure 5ab, *p* < 0.05). Additionally, compared to the control group, 10 µM ROT markedly reduced T-AOC activity (Figure 5c, *p* < 0.05) and increased H_2_O_2_ levels (Figure 5d, *p* < 0.05), with 0.3 mM EGT partially reversing this effect.

## 4. Discussion

The liquid preservation of boar semen is a critical component of AI, as the quality of sperm significantly influences the number of litters produced by sows [26,27,28]. However, with prolonged preservation, the ongoing metabolism of sperm results in nutrient depletion and the accumulation of metabolic by-products, which ultimately results in a decline in sperm quality [29]. In the liquid preservation of boar semen, the exogenous addition of antibiotics, antioxidants, sugars, and Chinese herbal extracts can inhibit sperm metabolism, reduce sperm motility, and decrease energy consumption, thereby facilitating the long-term preservation of boar semen [11,12]. EGT is a potent antioxidant that is commonly found in mammalian seminal plasma, with a concentration of 582 µM/mL in boar seminal plasma [15]. However, during the liquid preservation of boar semen at 17 °C, the concentration of EGT is notably low. This indicates that, compared to other antioxidants, external supplementation of EGT has a distinct advantage. Consequently, this study aimed to analyze the effect of EGT on the quality of boar sperm during *in vitro* liquid preservation and elucidate the underlying mechanisms.

The motility of sperm is a direct indicator of sperm quality [30]. Sperm with higher motility can swiftly reach the ampulla of the fallopian tube, the site of fertilization, thereby facilitating the fertilization process [31]. Research indicates that in the semen of male animals, parameters such as VAP, VSL, and VCL are directly correlated with the fertilization capability of sperm [32]. Additionally, sperm BCF has a positive correlation with sperm quality [33]. Furthermore, studies have also demonstrated that the VAP and VSL of ostrich sperm can directly reflect both the fertilization capability and fertility [34]. In this study, the results demonstrated that the addition of 0.3 mM EGT to the modified Modena extender significantly improved sperm motility parameters, including total motility, progressive motility, VAP, VSL, and VCL, during the liquid preservation of boar semen at 17 °C. In comparison to the 0.3 mM group, higher concentrations of EGT (0.6 mM) exhibited a reduction in sperm motility and kinetic parameters. This finding is consistent with the results reported by Bae et al., who demonstrated that 0.4 mM EGT significantly mitigated UVA-induced damage in human dermal fibroblasts [35]. Furthermore, Ko et al. found that 10 mM EGT could substantially decrease UVB-induced fibroblast damage [36]. These findings indicate that varying concentrations of EGT can mitigate different levels of cellular or sperm damage.

During the preservation of boar semen in liquid, prolonged preservation times result in increased ROS, which disrupts the balance between antioxidant and non-antioxidant enzyme systems in sperm. This disruption leads to oxidative stress and a subsequent decline in sperm quality. Numerous studies have demonstrated that EGT exhibits strong antioxidant properties, effectively reducing oxidative stress primarily by lowering ROS levels [37,38]. Moreover, research conducted by Sheridan et al. has shown that EGT significantly mitigates cell death induced by H_2_O_2_ [39]. Our study indicated that EGT significantly reduces oxidative damage to sperm by enhancing the activity of T-AOC and decreasing the levels of H_2_O_2_. This suggests that EGT can improve the motility parameters of sperm during the liquid preservation of boar semen by alleviating oxidative stress. These results are in agreement with the observed levels of EGT in boar seminal plasma [15].

Moreover, oxidative stress can severely compromise the structural integrity of sperm. The acrosome of sperm contains hydrolases, such as acrosin, hyaluronidase, and esterase, which are crucial for penetrating the oocyte membrane during fertilization [40]. This study demonstrated that EGT significantly enhances the acrosome integrity of sperm during liquid preservation of boar semen. Furthermore, the integrity of the sperm plasma membrane is essential for successful insemination [41,42]. Our results showed that, on days 3, 5, and 7, 0.3 mM EGT significantly improved plasma membrane integrity. This enhancement may be attributed to the gradual increase in oxidative stress levels over prolonged preservation periods, which leads to damage to the sperm acrosome and plasma membrane. In contrast, EGT mitigates this damage by reducing oxidative stress, thereby protecting the sperm acrosome and plasma membrane.

Mammalian sperm utilize ATP to maintain the stability of the intracellular environment and to facilitate various cellular processes, including motility, energy acquisition, hyperactivation, and the acrosomal response [43,44]. Insufficient ATP levels will result in fertilization failure. Our results indicated that EGT significantly enhanced both MMP and ATP levels in sperm during boar semen liquid preservation for 3 to 7 days. During the preservation of boar semen in liquid, an imbalance between the antioxidant system and ROS production primarily contributes to sperm damage [45]. Additionally, damage to the mitochondrial respiratory chain is a major source of ROS [46,47]. Meanwhile, studies have demonstrated that oxidative stress frequently correlates with a reduction in the expression of the MT-ND1 protein, a crucial component of the mitochondrial respiratory chain [48]. A previous study also indicated that pyrroloquinoline quinone not only decreased the ROS content in sperm but also enhanced the levels of mitochondrial proteins MT-ND1 [49]. Our results indicated that EGT significantly reduced ROS levels on day 5 of boar semen liquid preservation, providing preliminary evidence that EGT may enhance the preservation efficiency of boar semen at 17 °C by regulating the mitochondrial respiratory chain.

Numerous studies have demonstrated that ROT acts as an inhibitor of the mitochondrial respiratory chain [50,51,52]. To elucidate the mechanism by which EGT enhances the preservation efficiency of boar semen at 17 °C, we employed the mitochondrial respiratory chain inhibitor ROT to induce a model of sperm mitochondrial respiratory chain damage. The results indicated that treatment with 10 µM ROT for 3 h significantly reduced sperm motility and kinetic parameters. Consequently, we established a sperm mitochondrial respiratory chain damage model using 10 µM ROT for 3 h. Follow this, we co-treated sperm with 0.3 mM EGT and 10 µM ROT for 3 h, observing that EGT significantly restored the sperm motility and kinetic parameters. Additionally, this study revealed that EGT significantly mitigated the damage to the sperm acrosome and plasma membrane induced by ROT. Furthermore, EGT notably reduces the oxidative stress levels in sperm induced by ROT. Importantly, EGT reduced the excess ROS level induced by ROT. This further demonstrated that EGT mitigated ROS production by regulating the mitochondrial respiratory chain, thereby enhancing the preservation efficiency of boar semen at 17 °C. In-vivo fertility is a crucial method for verifying the efficacy of room-temperature preservation of boar semen [53]. This will be a primary focus of our subsequent research, aimed at determining whether the enhanced efficiency of room-temperature preservation of boar semen, achieved through EGT, can be effectively applied in the pig farming industry.

## 5. Conclusions

The results of this study demonstrated that 0.3 mM EGT enhanced the preservation efficiency of boar semen in liquid by modulating mitochondrial respiratory chains. This finding provides a theoretical reference for improving the liquid preservation efficiency of pig semen.

## Figures and Tables

**Table 1:** Effects of various concentrations of EGT on sperm motility and kinetic parameters.

Parameters	Groups (EGT)	Storage Time (Day)				
		0	1	3	5	7
Total motility/(%)	0 mM	88.06 ± 1.57	75.88 ± 1.23 ^a^	62.27 ± 1.06 ^a^	49.27 ± 1.74 ^a^	36.28 ± 0.36 ^a^
	0.15 mM	88.06 ± 1.57	78.45 ± 0.54 ^ab^	68.43 ± 0.71 ^b^	54.73 ± 0.68 ^b^	40.24 ± 1.39 ^b^
	0.3 mM	88.06 ± 1.57	82.07 ± 0.60 ^b^	73.58 ± 1.07 ^b^	59.31 ± 0.66 ^c^	48.58 ± 0.75 ^c^
	0.6 mM	88.06 ± 1.57	72.25 ± 1.72 ^a^	60.78 ± 0.86 ^a^	49.24 ± 1.65 ^a^	34.73 ± 1.45 ^a^
Progressive Motility/(%)	0 mM	78.87 ± 2.58	61.19 ± 1.23 ^ab^	44.23 ± 1.91 ^a^	35.21 ± 1.38 ^a^	23.22 ± 0.52 ^a^
	0.15 mM	78.87 ± 2.58	62.31 ± 0.54 ^a^	55.02 ± 0.86 ^b^	39.18 ± 0.55 ^b^	24.97 ± 0.65 ^ab^
	0.3 mM	78.87 ± 2.58	68.21 ± 0.60 ^c^	57.67 ± 0.62 ^b^	45.01 ± 1.05 ^c^	26.44 ± 0.25 ^b^
	0.6 mM	78.87 ± 2.58	59.06 ± 1.72 ^b^	44.72 ± 0.70 ^a^	39.77 ± 1.15 ^b^	25.98 ± 1.08 ^ab^
VAP/(μm/s)	0 mM	50.61 ± 2.10	34.59 ± 0.50 ^a^	31.34 ± 0.63 ^a^	16.47 ± 1.38 ^a^	18.35 ± 0.36 ^a^
	0.15 mM	50.61 ± 2.10	34.02 ± 0.33 ^a^	32.68 ± 1.54 ^a^	18.24 ± 0.55 ^a^	18.96 ± 0.39 ^a^
	0.3 mM	50.61 ± 2.10	42.65 ± 0.78 ^b^	39.88 ± 1.60 ^b^	30.58 ± 1.05 ^b^	25.44 ± 0.95 ^b^
	0.6 mM	50.61 ± 2.10	35.27 ± 0.65 ^a^	31.73 ± 1.13 ^a^	29.64 ± 1.15 ^b^	22.58 ± 1.55 ^b^
VSL/(μm/s)	0 mM	44.87 ± 1.15	36.91 ± 0.86 ^a^	32.34 ± 0.51 ^a^	13.51 ± 0.45 ^a^	12.74 ± 0.41 ^a^
	0.15 mM	44.87 ± 1.15	37.87 ± 0.40 ^a^	34.21 ± 0.92 ^a^	16.26 ± 0.77 ^b^	15.84 ± 0.77 ^b^
	0.3 mM	44.87 ± 1.15	41.71 ± 0.78 ^b^	38.80 ± 0.30 ^b^	28.74 ± 0.40 ^d^	23.82 ± 1.06 ^c^
	0.6 mM	44.87 ± 1.15	38.91 ± 0.66 ^a^	33.90 ± 0.53 ^a^	20.49 ± 0.49 ^c^	16.97 ± 0.53 ^b^
VCL/(μm/s)	0 mM	68.42 ± 1.66	51.12 ± 1.72 ^a^	36.24 ± 1.16 ^a^	21.40 ± 0.83 ^a^	14.95 ± 1.21 ^a^
	0.15 mM	68.42 ± 1.66	48.45 ± 1.64 ^a^	43.41 ± 0.83 ^a^	25.97 ± 1.10 ^a^	13.10 ± 1.23 ^a^
	0.3 mM	68.42 ± 1.66	60.59 ± 1.48 ^b^	45.40 ± 1.69 ^b^	35.54 ± 1.32 ^b^	23.25 ± 1.10 ^b^
	0.6 mM	68.42 ± 1.66	50.24 ± 1.56 ^a^	41.69 ± 1.49 ^a^	34.13 ± 0.93 ^b^	22.35 ± 1.58 ^b^

In the same column, values with different letter superscripts (a–c) represent a significant difference (*p* < 0.05), *n* = 3.

**Table 2:** Effects of different varieties of ROT on sperm sperm motility and kinetic parameters for 3 h.

Group (ROT)	Total Motility (%)	Progressive Motility (%)	VAP (μm/s)	VSL(μm/s)	VCL (μm/s)	BCF(Hz)
0 µM	95.02 ± 0.36 ^a^	87.25 ± 0.54 ^a^	57.13 ± 1.11 ^a^	54.80 ± 2.01 ^a^	81.37 ± 1.58 ^ab^	44.65 ± 1.23 ^a^
0.1 µM	94.33 ± 0.21 ^a^	89.08 ± 0.63 ^a^	59.58 ± 1.28 ^a^	46.51 ± 0.95 ^b^	84.86 ± 1.82 ^a^	46.53 ± 3.04 ^a^
1 µM	91.94 ± 0.17 ^a^	80.94 ± 0.36 ^b^	54.55 ± 0.90 ^a^	48.17 ± 0.94 ^b^	77.19 ± 1.13 ^b^	31.96 ± 1.34 ^b^
10 µM	82.24 ± 0.21 ^b^	61.96 ± 0.51 ^c^	42.88 ± 1.04 ^b^	39.31 ± 1.59 ^c^	61.07 ± 1.48 ^c^	29.31 ± 1.21 ^b^

In the same column, values with different letter superscripts (a–c) mean a significant difference (*p* < 0.05), *n* = 3.

**Table 3:** Effects of EGT and ROT co-treatment on boar sperm quality for 3 h.

Groups	Total Motility (%)	Progressive Motility (%)	VAP /(μm/s)	VSL /(μm/s)	VCL /(μm/s)	BCF (Hz)
Control	93.55 ± 0.22 ^a^	73.44 ± 1.59 ^a^	56.07 ± 0.98 ^a^	61.77 ± 1.30 ^a^	79.86 ± 1.39 ^a^	11.98 ± 0.51 ^a^
ROT	79.12 ± 0.29 ^c^	59.76 ± 1.67 ^b^	49.56 ± 0.86 ^b^	54.59 ± 1.04 ^b^	70.59 ± 1.22 ^b^	9.87 ± 0.42 ^b^
EGT + ROT	87.36 ± 0.28 ^b^	70.73 ± 0.67 ^a^	56.14 ± 1.05 ^a^	59.62 ± 0.64 ^a^	79.97 ± 1.49 ^a^	12.81 ± 0.44 ^a^

In the same column, values with different letter superscripts (a–b) represent a significant difference (*p* < 0.05), *n* = 3. The concentration employed for ROT is 10 µM, while the concentration utilized for EGT is 0.3 mM.

## Data Availability

The original contributions presented in the study are included in the article; further inquiries can be directed to the corresponding authors.

## Abbreviations

The following abbreviations are used in this manuscript:

EGT	Ergothioneine
MMP	Mitochondrial membrane potential
ATP	Adenosine triphosphate
MT-ND1	NADH dehydrogenase 1
ROT	Rotenone
ROS	Reactive oxygen species
SOD	Superoxide dismutase
GSH	Glutathione
MDA	Malondialdehyde
MCEAC	Management Committee of the Experimental Animal Center
CASA	Computer-assisted sperm analysis
VAP	Average path velocity
VSL	Average straight-line velocity
VCL	Average curvilinear velocity
BCF	Beat-cross frequency
STR	Straightness of path
PI	Propidium iodide
FITC-PNA	Fluorescein peanut agglutinin isothiocyanate
PBS	Phosphate-buffered saline
H2O2	Hydrogen peroxide
BSA	Bovine Serum Albumin

## References

[1] Johnson L.A., Weitze K.F., Fiser P., Maxwell W.M. (2000). Storage of boar semen. Anim. Reprod. Sci..

[2] Lopez Rodriguez A., Van Soom A., Arsenakis I., Maes D. (2017). Boar management and semen handling factors affect the quality of boar extended semen. Porc. Health Manag..

[3] Berean D., Blaga-Petrean A., Bogdan I., Bogdan S., Tamas-Krumpe O.M., Cimpean R., Pall E., Nap M.E., Bogdan L.M. (2023). Effect of L-arginine and eugenol on ram semen kinematic parameters and post thawed fertility rate after trans-cervical artificial insemination. Indian. J. Anim. Res..

[4] Tahara E.B., Navarete F.D.T., Kowaltowski A.J. (2009). Tissue-, substrate-, and site-specific characteristics of mitochondrial reactive oxygen species generation. Free. Radic. Biol. Med..

[5] Upadhyay V.R., Ramesh V., Dewry R.K., Yadav D.K., Ponraj P. (2022). Bimodal interplay of reactive oxygen and nitrogen species in physiology and pathophysiology of bovine sperm function. Theriogenology.

[6] Yeoman R.R., Jones W.D., Rizk B.M. (1998). Evidence for nitric oxide regulation of hamster sperm hyperactivation. J. Androl..

[7] Herrero M.B., Lamirande E., Gagnon C. (2003). Nitric Oxide is a Signaling Molecule in Spermatozoa. Curr. Pharm. Des..

[8] Sanocka D., Kurpisz M. (2004). Reactive oxygen species and sperm cells. Reprod. Biol. Endocrinol..

[9] Agarwal A., Said T.M. (2005). Oxidative stress, DNA damage and apoptosis in male infertility: A clinical approach. BJU Int..

[10] Zhu Z., Li R., Fan X., Lv Y., Zeng W., Longevity C. (2019). Resveratrol Improves Boar Sperm Quality via 5′AMP-Activated Protein Kinase Activation during Cryopreservation. Oxid. Med. Cell. Longev..

[11] Iakovlev T.E. (1970). Effect of antibiotics on the quantity of sperm of bulls and boars. Veterinariia.

[12] Li D., Zhang W., Tian X., He Y., Xiao Z., Zhao X., Lin F., Renrang D., Gongshe Y., Taiyong Y. (2022). Hydroxytyrosol effectively improves the quality of pig sperm at 17 degrees C. Theriogenology.

[13] Koppers A.J., De I.G.N., Finnie J.M., McLaughlin E.A., John A.R. (2008). Significance of Mitochondrial Reactive Oxygen Species in the Generation of Oxidative Stress in Spermatozoa. J. Clin. Endocrinol. Metab..

[14] Nielsen J. (2022). Bioactive metabolites: The double-edged sword in your food. Cell.

[15] Nikodemus D., Lazic D., Bach M., Bauer T., Pfeiffer C., Wiltzer L., Lain E., Schömig E., Gründemann D. (2011). Paramount levels of ergothioneine transporter SLC22A4 mRNA in boar seminal vesicles and cross-species analysis of ergothioneine and glutathione in seminal plasma. J. Physiol. Pharmacol..

[16] Salama S.A., Omar H.A. (2021). Modulating NF-κB, MAPK, and PI3K/AKT signaling by ergothioneine attenuates iron overload-induced hepatocellular injury in rats. J. Biochem. Mol. Toxicol..

[17] Ishimoto T., Masuo Y., Kato Y., Nakamichi N. (2019). Ergothioneine-induced neuronal differentiation is mediated through activation of S6K1 and neurotrophin 4/5-TrkB signaling in murine neural stem cells. Cell. Signal..

[18] Li J., Dong Y., Wang H., Zhang Q., Guo Q., Li Y. (2025). The Cryoprotectant Effects of Safffower Polysaccharides on the Quality of Frozen-Thawed Boar Sperm. Animals.

[19] Li Y., Wang H., Wang S., Zhang Q., Zhang H., Li T., Wang Q., Guo M., Feng H., Song Y. (2023). Methylprednisolone improves the quality of liquid preserved boar spermatozoa in vitro and reduces polymorphonuclear neutrophil chemotaxis and phagocytosis. Front. Vet. Sci..

[20] Zhu Z., Umehara T., Okazaki T., Goto M., Fujita Y., Hoque S.A.M., Kawai T., Zeng W., Shimada M. (2019). Gene Expression and Protein Synthesis in Mitochondria Enhance the Duration of High-Speed Linear Motility in Boar Sperm. Front. Physiol..

[21] Nadaf S.M., Ramesh V., Mech M., Haider Khan M., Ahmed F.A., Ponraj P., Mitra A. (2022). Comparative ejaculatory response, fresh and frozen semen quality and fertility to artificial vagina vs electroejaculation method of semen collection in mithun (*Bos frontalis*) bulls. Andrologia.

[22] Ma H., Quan F., Chen D., Zhang B., Zhang Y. (2010). Alterations in mitochondrial function and spermatozoal motility in goat spermatozoa following incubation with a human lysozyme plasmid. Anim. Reprod. Sci..

[23] Guo H.T., Wang J.R., Sun L.Z., Jin X.H., Shi X.Y., Lin J.Y., Yue S.L., Zhou J.B. (2021). Effects of astaxanthin on plasma membrane function and fertility of boar sperm during cryopreservation. Theriogenology.

[24] Aboagla M.E., Maeda T. (2011). Arbutin’s suppression of cryodamage in goat sperm and its mechanism of cryoprotection. Theriogenology.

[25] Zhu Z., Kawai T., Umehara T., Hoque S.A.M., Zeng W., Shimada M. (2019). Negative effects of ROS generated during linear sperm motility on gene expression and ATP generation in boar sperm mitochondria. Free Radic. Biol. Med..

[26] Rodríguez A.L., Rijsselaere T., Vyt P., Soom A.V., Maes D. (2012). Effect of Dilution Temperature on Boar Semen Quality. Reprod. Domest. Anim..

[27] Pavaneli A.P.P., Passarelli M.D.S., Freitas D., Vieira F., Ravagnani G.M., Torres M.A., Martins S.M.M.K., Yeste M., Andrade A.F.C.D. (2019). Removal of seminal plasma prior to liquid storage of boar spermatozoa: A practice that can improve their fertilizing ability. Theriogenology.

[28] Lee S., Iwasaki Y., Shikina S., Yoshizaki G. (2013). Generation of functional eggs and sperm from cryopreserved whole testes. Proc. Natl. Acad. Sci. USA.

[29] Karunakaran M., Chakurkar E.B., Ratnakaran U., Naik P.K., Mondal M., Mondal A., Singh N.P. (2016). Characteristics of boar semen preserved at liquid state. J. Appl. Anim. Res..

[30] Lewandowska E., Wesierski D., Mazur-Milecka M., Liss J., Jezierska A. (2023). Ensembling noisy segmentation masks of blurred sperm images. Comput. Biol. Med..

[31] Van de Hoek M., Rickard J.P., de Graaf S.P. (2022). Motility Assessment of Ram Spermatozoa. Biology.

[32] Hirano Y., Shibahara H., Obara H., Suzuki T., Takamizawa S., Yamaguchi C., Tsunoda H., Sato I. (2001). Relationships between Sperm Motility Characteristics Assessed by the Computer-Aided Sperm Analysis (CASA) and Fertilization Rates in Vitro. J. Assist. Reprod. Genet..

[33] Liu D.Y., Clarke G.N., Baker H.W. (1991). Relationship between Sperm Motility Assessed with the Hamilton-Thorn Motility Analyzer and Fertilization Rates in Vitro. J. Androl..

[34] Muvhali P.T., Bonato M., Malecki I.A., Cloete S.W.P. (2022). Mass Sperm Motility Is Correlated to Sperm Motility as Measured by Computer-Aided Sperm Analysis (CASA) Technology in Farmed Ostriches. Animals.

[35] Bae J.T., Lee C.H., Lee G.S., Kim J.H., Hong J.T. (2019). Glycation inhibitory and antioxidative activities of ergothioneine. J. Soc. Cosmet. Sci. Korea.

[36] Ko H.J., Kim J., Ahn M., Kim J.H., Lee G.S., Shin T. (2021). Ergothioneine alleviates senescence of fibroblasts induced by UVB damage of keratinocytes via activation of the Nrf2/HO-1 pathway and HSP70 in keratinocytes. Exp. Cell Res..

[37] Li R.W.S., Yang C., Sit A.S.M., Kwan Y.W., Lee S.M.Y., Hoi M.P.M., Chan S.W., Hausman M., Vanhoutte P.M., Leung G.P.H. (2014). Uptake and protective effects of ergothioneine in human endothelial cells. J. Pharmacol. Exp. Ther..

[38] Behof W.J., Whitmore C.A., Haynes J.R., Rosenberg A.J., Tantawy M.N., Peterson T.E., Harrison F.E., Beelman R.B., Pham W. (2021). A novel antioxidant ergothioneine PET radioligand for in vivo imaging applications. Sci. Rep..

[39] Aruoma O.I., Spencer J.P., Mahmood N. (1999). Protection against oxidative damage and cell death by the natural antioxidant ergothioneine. Food. Chem. Toxicol..

[40] Olaciregui M., Luño V., Domingo P., González N., Gil L. (2017). In vitro developmental ability of ovine oocytes following intracytoplasmic injection with freeze-dried spermatozoa. Sci. Rep..

[41] Berger T., Anderson D.L., Penedo M.C.T. (1996). Porcine sperm fertilizing potential in relationship to sperm functional capacities. Anim. Reprod. Sci..

[42] Sutkeviciene N., Riskeviciene V., Januskauskas A., Zilinskas H., Andersson M. (2009). Assessment of sperm quality traits in relation to fertility in boar semen. Acta. Vet. Scand..

[43] Mannowetz N., Wandernoth P.M., Wennemuth G. (2012). Glucose is a pH-Dependent Motor for Sperm Beat Frequency during Early Activation. PLoS ONE.

[44] Gnaiger E. (2001). Bioenergetics at low oxygen: Dependence of respiration and phosphorylation on oxygen and adenosine diphosphate supply. Respir. Physiol..

[45] Ko E.Y., Sabanegh E.S., Agarwal A. (2014). Male infertility testing: Reactive oxygen species and antioxidant capacity. Fertil. Steril..

[46] Graziewicz M.A., Day B.J., Copeland W.C. (2002). The mitochondrial DNA polymerase as a target of oxidative damage. Nucleic. Acids. Res..

[47] Tan E.C.T., Janssen A.J.M., Roestenberg P., Heuvel L.P.V.D., Rodenburg R.J.T. (2011). Mitochondrial dysfunction in muscle tissue of complex regional pain syndrome type I patients. Eur. J. Pain..

[48] Xie X., Le L., Fan Y., Lv L., Zhang J. (2012). Autophagy is induced through the ROS-TP53-DRAM1 pathway in response to mitochondrial protein synthesis inhibition. Autophagy.

[49] Zhu Z., Li W., Yang Q., Zhao H., Zhang W., Adetunji A.O., Hoque S.A.M., Kou X., Min L. (2024). Pyrroloquinoline Quinone Improves Ram Sperm Quality through Its Antioxidative Ability during Storage at 4 °C. Antioxidants..

[50] Heo G., Sun M.H., Jiang W.J., Li X.H., Lee S.H., Guo J., Zhou D.J., Cui X.S. (2022). Rotenone causes mitochondrial dysfunction and prevents maturation in porcine oocytes. PLoS ONE.

[51] Plaza Dávila M., Bucci D., Galeati G., Peña F.J., Mari G., Giaretta E., Tamanini C., Spinaci M. (2015). Epigallocatechin-3-Gallate (EGCG) Reduces Rotenone Effect on Stallion Sperm-Zona Pellucida Heterologous Binding. Reprod. Domest. Anim..

[52] Rogers B.J., Ueno M., Yanagimachi R. (1977). Inhibition of hamster sperm acrosome reaction and fertilization by oligomycin, antimycin A, and rotenone. J. Exp. Zool..

[53] Singh M., Mollier R.T., Kumar D., Katiyar R., Chamuah J.K., Kumar S., Chaudhary J.K., Deori S., Kalita H., Mishra V.K. (2024). Temporal effect of flaxseed oil in boar’s diet on semen quality, antioxidant status and in-vivo fertility under hot humid sub-tropical condition. Sci. Rep..

